# Clinical Characteristics and Management of Patients Admitted to the Supportive Care Clinic and Predisposing Factors of Unplanned Hospital Readmission: Single-Center Experience

**DOI:** 10.3390/jcm14082679

**Published:** 2025-04-14

**Authors:** Onur Baş, Mert Tokatlı, Naciye Güdük, Dilara Erdoğan, Nur Evşan Boyraz, Çağla Çengelci, Deniz Can Guven, Ömer Dizdar, Fatma Alev Türker, Sercan Aksoy

**Affiliations:** 1 Division of Medical Oncology, Department of Internal Medicine, Faculty of Medicine, Hacettepe University, Sihhiye, 06100 Ankara, Turkey; denizcguven@hotmail.com (D.C.G.); dromerdizdar@gmail.com (Ö.D.); aturker@hacettepe.edu.tr (F.A.T.); saksoy07@yahoo.com (S.A.); 2 Department of Internal Medicine, Faculty of Medicine, Hacettepe University, Sihhiye, 06100 Ankara, Turkey; merttokatli17@gmail.com (M.T.); naciyeguduk@hotmail.com (N.G.);

**Keywords:** supportive care clinic, unplanned hospital readmission, outpatient, inpatient

## Abstract

**Background:** It is well known that supportive care clinics are vital in medical oncology practice. This study aims to present a portrait of the supportive care clinic of a tertiary cancer center in a low-middle-income country. **Methods:** This study included patients admitted to our supportive care clinic between January 2019 and December 2023. This study included patients who attended the supportive care clinic. For patients who were readmitted more than once throughout the study period, only the first readmission was included in the analysis. The primary outcome of this study was a better understanding of the risk factors associated with hospital readmissions in cancer patients, which could lead to improved patient outcomes. In addition, the secondary objective was to identify the characteristics of patients and clinical decisions regarding follow-ups as inpatients or outpatients referred to the supportive care clinic. **Results:** This study included 477 patients; 300 (62.9%) were directed to oncology inpatient care, while 177 (37.1%) were treated as outpatients. The most common diagnoses were lung cancer (20.5%), colorectal cancer (13.6%), and breast cancer (8.4%). Most patients (71.5%) were evaluated for symptom palliation. In multivariate analysis, metastatic disease (OR: 2.52 95% CI 1.48–4.29 *p* = 0.001), Eastern Cooperative Oncology Group (ECOG) performance status (OR: 1.58 95% CI 1.04–2.42 *p* = 0.034), and a decrease in albumin levels (OR: 0.42 95% CI 0.29–0.61 < 0.001) were significantly related to hospitalization. In univariate analyses, albumin level (*p* < 0.001), disease stage (*p* = 0.007), and ECOG performance status (*p* = 0.025) were statistically associated with unplanned hospital readmission. Among these factors, a decrease in albumin levels was significantly associated with the outcome, with an odds ratio of 0.54 (95% CI 0.39–0.75, *p* < 0.001), indicating a protective effect of higher albumin levels. In univariate analyses, sex (*p* = 0.016), cancer treatment type (*p* = 0.010), albumin level (*p* < 0.001), disease stage (*p* < 0.001), unplanned hospital readmission (*p* < 0.001), ECOG performance status (*p* < 0.001), and hemoglobin (*p* = 0.008) were statistically related to overall survival. Among these factors, sex (HR: 1.28 95% CI 1.03–1.59 *p* = 0.025), a decrease in albumin levels (HR: 0.67 95% CI 0.56–0.82 *p* < 0.001), disease stage (HR: 1.52 95% CI 1.11–2.09 *p* = 0.008), unplanned hospital readmission (HR: 1.30 95% CI 1.03–1.63 *p* = 0.027), and ECOG performance status (HR: 3.45 95% CI 2.68–4.45 *p* < 0.001) remained significant in the multivariate analysis. **Conclusions:** This study shows that supportive care clinics are a key element of patient care. Early evaluation of patients in supportive care clinics may aid clinicians in identifying high-risk patients who may require closer follow-up or inpatient care. Several factors were identified as predisposing to hospitalization, unplanned hospital readmission, and overall survival. Further prospective studies are needed to determine the risk factors associated with hospitalization, readmission, and overall survival.

## 1. Introduction

Medical oncology departments have traditionally relied on inpatient ward beds for the majority of their treatments. This is due to the significant treatment-related toxicities, including nausea, pain, decreased oral feeding, infections, and the need for interventional procedures. However, there has been a growing trend for selected patients to be treated in the outpatient setting, both in non-palliative and palliative settings [1]. This has been carried out both during active treatment and the follow-up period after cessation of treatment.

The term “supportive care” is a general one, with a primary focus on the management of symptoms and the enhancement of quality of life. An illustrative example of this approach is the management of adverse effects of chemotherapy, such as nausea and weight loss, in patients undergoing curative treatment. In contrast, “palliative care” constitutes a specialized form of care that provides support to patients in the final stages of their lives. Despite the presence of significant conceptual differences, these terms are used interchangeably.

In low-middle-income countries (LMICs), there may be a shortage of personnel and/or equipment in emergency rooms and oncology wards like in our country. Consequently, supportive care clinics assume a pivotal role in the management of oncological care, serving as primary access points for patients. Supportive care clinics play an important role in enhancing outpatient care services and facilitating the early diagnosis of emergencies needing immediate inpatient care, such as severe infections and respiratory distress.

Thirty-day readmission is defined as unplanned readmission to the hospital within thirty days of discharge. Patients with cancer frequently experience unplanned readmission to the hospital, often because of a need for palliative or supportive care, or the occurrence of more severe complications necessitating interventional procedures such as thoracentesis, paracentesis, and others [2,3]. Furthermore, readmission rates within the cancer population exhibit a higher rate than those of the general patient population [4]. This phenomenon leads clinicians and healthcare providers to investigate the underlying factors contributing to unplanned hospital readmission [5]. Additionally, unplanned readmissions to the hospital represent a significant medical problem that is utilized as a quality indicator analogous to the monitoring of infection rates [6]. Readmissions are primarily associated with reduced quality of life and increased morbidity [7,8]. Therefore, assessing the frequency of readmissions and the underlying factors is crucial to identifying high-risk patients and preventing readmissions [9].

In this study, we aimed to demonstrate the clinical characteristics and management of patients admitted to the supportive care clinic in an LMIC and the predisposing factors of readmission and parameters related to overall survival.

## 2. Materials and Methods

### 2.1. Patients

We conducted a retrospective study of patients admitted to the supportive care clinic at Hacettepe University Hospital between January 2019 and December 2023. This study included patients who attended the supportive care clinic, while those under the age of eighteen and those without a definitive cancer diagnosis were excluded from the study. For patients who were readmitted more than once throughout the study period, only the first readmission was included in the analysis.

### 2.2. Definition and Purpose of Supportive Care Clinic

The supportive care clinic functions as the primary contact point for patients, who can be admitted directly without the need for an appointment. The unit includes one medical oncologist, one radiation oncologist, one internist, three nurses (one specifically for pain management), and one oncology dietician.

The primary objective of this clinic is to reduce the waiting time of patients in emergency rooms and to identify high-risk patients who need immediate inpatient care or intervention.

### 2.3. Definition of Unplanned Hospital Readmission

The term “unplanned hospital readmission” was defined as a readmission to the hospital within 30 days of discharge (for the inpatient group) or of the initial admission to the supportive care clinic (for the outpatient group).

### 2.4. Data Collection

We collected detailed information on every patient from their medical records, which included their age, medical history, any additional health conditions, type of cancer, results from their initial laboratory tests, ongoing anti-cancer treatments (whether systemic treatment or radiotherapy), medications they regularly use, and the reasons for their admissions.

### 2.5. Outcomes

The primary outcome of this study was a better understanding of the risk factors associated with hospital readmissions in cancer patients, which could lead to improved patient outcomes. In addition, the secondary objective was to identify the characteristics of patients and clinical decisions regarding follow-up as inpatients or outpatients referred to the supportive care clinic. Finally, the goal was to demonstrate which covariates were associated with overall survival.

### 2.6. Statistical Analysis

We used IBM-SPSS version 24 for analysis. The data were presented in two forms: firstly, as the median with interquartile range (IQR) for continuous variables, and secondly, as the number of patients with percentages for categorical variables. Groups were compared using the chi-square test for categorical variables. We used the Kolmogorov–Smirnov test to check the normality of the data distribution. T-tests were used for normally distributed data. Overall survival (OS) time is specified as the duration from the initiation of treatment to either the final visit or mortality. We performed survival analyses using Kaplan–Meier methods and compared survival times between different groups with the log-rank test. To assess the impact of multiple covariates on survival, we conducted a multivariate analysis using Cox’s proportional hazards model. Additionally, we performed logistic regression analysis to identify important factors associated with hospitalization (Supplementary Tables S1 and S2). Variables with a significance level of α = 0.20 in univariable analysis were included in the multivariable model, along with other relevant factors identified from previous research.

## 3. Results

### 3.1. Baseline Characteristics

This study included 477 patients; 300 (62.9%) were directed to oncology inpatient care, while 177 (37.1%) were treated as outpatients. Table 1 presents the demographic and clinicopathologic characteristics of the study population. The median age was 60 years (interquartile range [IQR] = 51–67). In addition, 240 (50.3%) were female and 237 (49.7%) were male. The most common diagnoses were lung cancer (20.5%), colorectal cancer (13.6%), and breast cancer (8.4%). Additionally, 388 (81.3%) patients had metastatic disease, and 246 (51.6%) had an ECOG performance status ≥ 2. Most patients (71.5%) were evaluated for symptom palliation, including nausea, pain, dyspnea, decreased oral feeding, and fatigue. The median follow-up from admission to the outpatient clinic to the last control was 100 days (IQR = 30–303). The median length of hospital stay was 9 days (IQR= 4–16).

In multivariate analysis, metastatic disease (OR: 2.52 95% CI 1.48–4.29 *p* = 0.001), Eastern Cooperative Oncology Group (ECOG) performance status (OR: 1.58 95% CI 1.04–2.42 *p* = 0.034), and lower albumin levels (OR: 0.42 95% CI 0.29–0.61 < 0.001) were significantly related to hospitalization (Table 1).

### 3.2. Unplanned Hospital Readmissions

**Figure 1:**
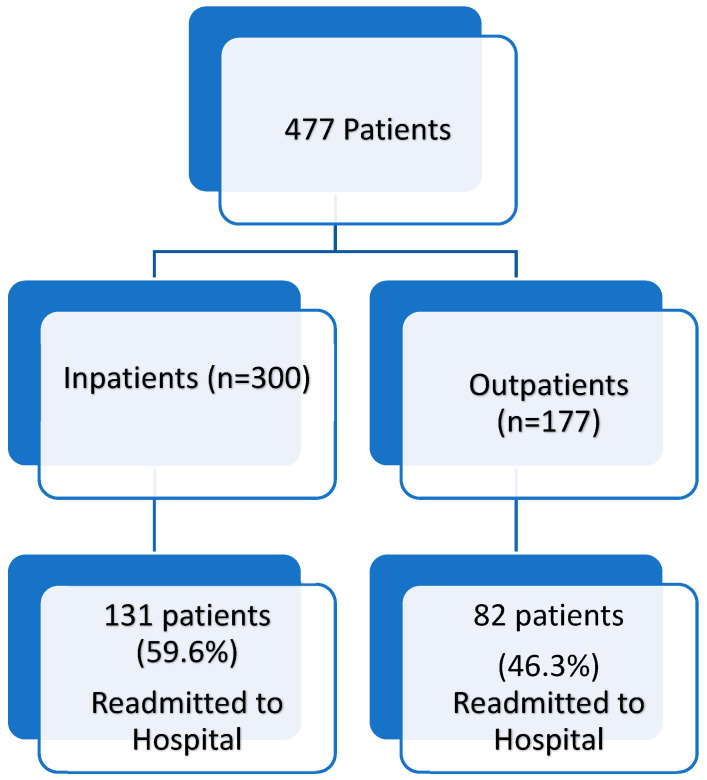
Readmission rates of patients admitted to the supportive care clinic.

Unplanned hospital readmission rates of patients admitted to our hospital are shown in Figure 1.

In univariate analyses, albumin levels (*p* < 0.001), disease stage (*p* = 0.007), and ECOG performance status (*p* = 0.025) were statistically associated with unplanned hospital readmission. Among these factors, a decrease in albumin levels was significantly associated with the outcome, with an odds ratio of 0.54 (95% CI 0.39–0.75, *p* < 0.001), indicating a protective effect of higher albumin levels (Table 2a,b).

### 3.3. Overall Survival

In univariate analyses, sex (*p* = 0.016), cancer treatment type (*p* = 0.010), albumin level (*p* < 0.001), disease stage (*p* < 0.001), unplanned hospital readmission (*p* < 0.001), ECOG performance status (*p* < 0.001), and hemoglobin (*p* = 0.008) were statistically related to overall survival. Among these factors, sex (1.28 95% CI 1.03–1.59 *p* = 0.025), a decrease in albumin levels (0.67 95% CI 0.56–0.82 *p* < 0.001), disease stage (1.52 95% CI 1.11–2.09 *p* = 0.008), unplanned hospital readmission (1.30 95% CI 1.03–1.63 *p* = 0.027), and ECOG performance status (3.45 95% CI 2.68–4.45 *p* < 0.001) remained significant in multivariate analysis (Table 3a,b).

## 4. Discussion

In this study, 300 patients (62.9%) required hospitalization, while 177 patients (37.1%) were managed as outpatients. Metastatic disease, ECOG performance status, and albumin level were significantly related to hospitalization. A total of 187 patients (39.2%) from the inpatient group and 82 patients (46.3%) from the outpatient group had unplanned hospital readmission. Albumin levels were the primary predictor of unplanned hospital readmission. Sex, albumin level, disease stage, unplanned hospital readmission, and ECOG performance status were identified as the main predictors of overall survival.

Supportive care clinics play a critical role in the care of cancer patients. In our institution, the supportive care clinic serves as the primary point of contact for patients, even before the emergency service. Therefore, patients must be referred to suitable care services promptly [10]. There are two principal reasons for this. Firstly, it helps to identify patients who require supportive or palliative care. Secondly, it can improve patient outcomes [11,12,13,14]. In Ghana, an LMIC, it was demonstrated that timely referral to palliative/supportive care improved symptom management and psychological support [15]. In our institution, we use similar criteria to the international consensus [16] for palliative care referral. Overall, it is crucial to evaluate patients promptly to determine whether they require inpatient or outpatient follow-up, and if further supportive or palliative care is necessary, thus avoiding unnecessary emergency department visits [17,18]. Further research is needed to understand the impact of supportive care on patients and healthcare systems.

Previous studies conducted in medical oncology services reported lower readmission rates than ours [3,9,19]. Although these studies mainly include similar populations to ours, predictor factors of unplanned hospital readmission are different [20,21,22]. For instance, metastatic disease and albumin levels were identified as significant predisposing factors for readmissions in other studies. In our study, there was a trend for increased readmission risk in patients with metastatic disease (*p* = 0.155) and albumin levels (*p* = 0.055). Similarly, although polypharmacy is a known risk factor for unplanned hospital readmission, our study found no relationship between polypharmacy and unplanned hospital readmission (*p* = 0.924) [23]. This may be due to the relatively small sample size, the timing of the study, and the heterogeneity of the study population. Therefore, we believe that institution-based studies are better for understanding the reasons behind readmissions. However, prospective studies are still needed to define readmission risk and predictive factors.

In many LMICs, due to a lack of facilities, there are not enough supportive care clinics available for specific cancer types. Therefore, we include all cancer patients referred to our clinic. However, it should be noted that palliative care for lung cancers may differ from that for gastrointestinal cancers. On the other hand, most of the patients referred to our supportive care clinic had metastatic disease and were not being treated curatively. Consequently, the management and characterization of these patients were analogous, particularly with consideration of palliative care modalities such as pain management, cachexia treatment, and supportive interventions. Similar studies in this area have also looked at all patients with different types of cancer [3,24,25]. Overall, although this may seem like a major confounding factor, our study reflects a real-world setting, which is valuable in demonstrating risk factors for unplanned hospital readmissions.

Our findings indicate that several factors, including unplanned readmission, hemoglobin, and albumin level, are associated with overall survival [26,27,28,29]. Similar to the literature (mainly from the USA), our data strongly support the idea that unplanned hospital readmission patients had significantly decreased survival [30,31]. Both albumin level and hemoglobin are closely linked to the nutritional status of patients [32,33,34]. In addition to nutritional status, hemodynamic status—which is related to the general health status of patients—also needs to be considered. Research has demonstrated that perfusion status is associated with survival outcomes, especially in elderly or fragile patients [35]. Occupational therapy services are also important. Therefore, we recommended that every patient should receive dietary consultation/nutritional support, which should be reassessed at follow-up clinic visits. In light of the previously mentioned points, it can be stated that working with oncology dieticians may improve the quality of life and survival of patients.

Preventing unplanned hospital readmissions may increase patients’ quality of life and overall survival. It is recommended that advanced care planning and palliative care consultations be employed to prevent unplanned hospital readmissions. Although occupational therapy services are not readily available in LMICs, these therapies improve patient outcomes and reduce hospital readmissions [36]. Furthermore, post-discharge rehabilitation and follow-up programs may improve patients’ quality of life with preventable readmissions [37]. The inclusion of oncology psychologists, occupational therapy practitioners, and religious officials in the planning of advanced and end-of-life care may be a strategy to enhance the quality of palliative care.

This study has several limitations. Firstly, the results may have been influenced by the retrospective nature of the study and the relatively small sample size. The single-center study design may have increased the risk of selection bias. Secondly, the socioeconomic and discharge statuses of patients were not included, which were important risk factors for readmission. Thirdly, the patient population included various cancers with different treatment types. Finally, readmissions to other institutions were not evaluated during the specified period. Therefore, the results of this study should be interpreted with caution. Despite these limitations, our findings indicated the presence of several predisposing factors that could help clinicians in determining inpatient or outpatient follow-up as well as the prediction of survival.

## 5. Conclusions

A significant proportion of oncology patients required hospitalization, with a high prevalence of early readmission. Several factors were identified as predisposing to hospitalization, readmission, and overall survival. Further prospective studies are required to identify the risk factors associated with hospitalization, readmission, and overall survival.

## Figures and Tables

**Table 1:** Baseline characteristics of patients were grouped as follows: treated as outpatients or inpatients. Univariate and logistic regression analyses were also performed to establish the differences between the groups.

Characteristic	Treated as Outpatients (*n* = 177)	Treated as Inpatients (*n* = 300)	Univariate Analysis *p*-Value	Multivariate Analysis OR (95% CI) *p* Value
Median Age (IQR)	59 (52–68)	61 (51–67)	0.909	(-)
Sex			0.059	0.421
Male	78 (44.1%)	159 (53%)		
Female	99 (55.9%)	141 (47%)		
Primary Diagnosis			0.203	(-)
Other * GI Cancers	34 (19.2%)	66 (22.0%)		
Colorectal Cancers	21 (11.9%)	44 (14.7%)		
Lung Cancer	34 (19.2%)	64 (21.3)		
Breast Cancer	17 (9.6%)	25 (8.3%)		
Other ** Cancers	32 (18.1%)	52 (17.3%)		
Gynecologic Cancers	26 (14.7%)	31 (10.3%)		
Genitourinary Cancers	13 (7.3%)	18 (6.0%)		
Disease Stage			<0.001	2.52 (95% CI 1.48–4.29) *p* = 0.001
Metastatic	127 (71.8%)	261 (87%)		
Non-Metastatic	50 (28.2%)	39 (13%)		
Number of Comorbidities			0.069	0.316
<3	146 (82.5%)	226 (75.3%)		
≥3	31 (17.5%)	74 (24.7%)		
ECOG Performance Status			<0.001	1.58 (95% CI 1.04–2.42) *p* = 0.034
<2	98 (55.4%)	105 (35%)		
≥2	79 (44.6%)	195 (65%)		
Number of Regularly Used Drugs			0.040	0.096
<5	148 (83.6%)	223 (74.3%)		
≥5	29 (16.4%)	72 (24.0%)		
Unknown	0	5 (1.7%)		
Type of Cancer Treatment			0.779	(-)
Chemotherapy	110 (62.1%)	193 (64.3%)		
Radiotherapy	16 (9.0%)	16 (5.4%)		
Others ***	31 (17.5)	46 (15.3%)		
No Active Treatment	20 (11.3%)	45 (15%)		
Indication for Follow-Up			0.073	0.152
Symptom Palliation	135 (76.3%)	206 (68.7%)		
Infection and/or Interventional Procedure	42 (23.7%)	95 (31.3%)		
Hemoglobin Level				
Mean (sd) (g/dL)	10.7 (2.1)	10.2 (2.2)	0.03	0.89
Albumin Level				
Mean (sd) (g/dL)	3.39 (0.59)	3.03 (0.58)	<0.001	0.42 (95% CI 0.29–0.61) *p* <0.001

* Gastric cancer, esophagus cancer, pancreas cancer, cholangiocarcinoma, and hepatocellular carcinoma, ** Lymphoma, sarcoma, malignant melanoma, unknown primary, head and neck cancer, and glioblastoma multiforme. *** Tyrosine kinase inhibitor, immunotherapy, and antibody–drug conjugate. IQR: interquartile range; ECOG: Eastern Cooperative Oncology Group; GI: gastrointestinal.

**Table 2:** (a) Univariate analysis of covariates associated with unplanned hospital readmission. (b) Multivariate analysis of covariates associated with unplanned hospital readmission.

**(a)**								
**95% CI**								
**Variables**	**B**	**S.E.**	**Wald**	**df**	***p*-Value**	**OR**	**Lower**	**Upper**
Age (Years)	0.011	0.008	2.11	1	0.146	1.01	0.996	1.03
Sex	−0.191	0.188	1.03	1	0.309	0.83	0.57	1.20
Male								
Female								
Type of Cancer Treatment								
Chemotherapy (Ref)								
Radiotherapy	−0.098	0.379	0.067	1	0.102	0.91	0.43	1.91
Others *	−0.452	0.257	3.093	1		0.64	0.39	1.05
No Active Treatment	0.407	0.297	1.872	1		1.50	0.84	2.69
Albumin (g/dL)	−0.687	0.165	17.38	1	<0.001	0.50	0.36	0.70
Disease Stage	0.639	0.237	7.28	1	0.007	1.89	1.20	3.02
Metastatic								
Non-Metastatic								
ECOG Performance Status	0.426	0.190	5.03	1	0.025	1.53	1.06	2.22
<2								
≥2								
Hemoglobin (g/dL)	−0.006	0.043	0.022	1	0.88	0.99	0.91	1.08
**(b)**								
**95% CI**								
**Variables**	**B**	**S.E.**	**Wald**	**df**	***p*-Value**	**OR**	**Lower**	**Upper**
Age (Years)	0.008	0.008	0.882	1	0.345	1.008	0.99	1.02
Type of Cancer Treatment								
Chemotherapy (Ref)								
Radiotherapy	0.017	0.430	0.002	1	0.171	1.02	0.44	2.35
Others *	−0.434	0.275	2.48	1		0.66	0.39	1.13
No Active Treatment	0.382	0.321	1.42	1		1.54	0.83	2.87
Albumin (g/dL)	−0.614	0.169	13.18	1	<0.001	0.54	0.39	0.75
Disease Stage	0.401	0.259	2.40	1	0.121	0.67	0.40	1.11
Metastatic								
Non-Metastatic								
ECOG Performance Status	0.149	0.222	0.45	1	0.502	0.87	0.57	1.33
<2								
≥2								
Constant	2.405	0.553	18.91	1	<0.001	11.08		

Eastern Cooperative Oncology Group: ECOG; B: coefficients; S.E.: standard error; df: degrees of freedom; OR: odds ratio; CI, 95%: confidence interval. * Tyrosine kinase inhibitor, immunotherapy, and antibody–drug conjugate.

**Table 3:** (a) Univariate analysis of covariates associated with overall survival. (b) Multivariate analysis of covariates associated with overall survival.

**(a)**								
**95% CI**								
**Variables**	**B**	**S.E.**	**Wald**	**df**	***p*-Value**	**HR**	**Lower**	**Upper**
Age (Years)	−0.001	0.004	0.07	1	0.798	0.99	0.99	1.007
Sex	0.266	0.108	6.07	1	0.016	0.77	0.62	0.95
Male								
Female								
Number of Regularly Used Drugs	0.063	0.131	0.23	1	0.630	1.07	0.82	1.38
<5								
≥5								
Number of Comorbidities	−0.035	0.133	0.071	1	0.770	0.97	0.74	1.25
<3								
≥3								
Type of Cancer Treatment								
Chemotherapy (Ref)								
Radiotherapy	−0.411	0.239	2.95	1	0.010	0.66	0.42	1.06
Others *	<0.001	0.151	<0.001	1		1.00	0.75	1.34
No Active Treatment	0.362	0.160	5.11	1		1.44	1.05	1.96
Albumin (g/dL)	−0.720	0.091	62.92	1	<0.001	0.43	0.36	0.51
Disease Stage	0.668	0.155	18.48	1	<0.001	1.95	1.44	2.65
Metastatic								
Non-Metastatic								
ECOG Performance Status	1.377	0.124	124.23	1	<0.001	3.96	3.11	5.05
<2								
≥2								
Unplanned Hospital Readmission	0.436	0.114	14.56	1	<0.001	1.55	1.24	1.93
Yes								
No								
Hemoglobin (Mean) (g/dL)	−0.068	0.026	7.00	1	0.008	0.93	0.89	0.98
Indication for Follow-Up	−0.091	0.122	0.564	1	0.45	0.913	0.72	1.16
Symptom Palliation								
Infection and/or Interventional Procedure								
**(b)**								
**95% CI**								
**Variables**	**B**	**S.E.**	**Wald**	**df**	***p*-Value**	**HR**	**Lower**	**Upper**
Sex	0.249	0.111	5.03	1	0.025	1.28	1.03	1.59
Male								
Female								
Type of Cancer Treatment								
Chemotherapy (Ref)								
Radiotherapy	−0.300	0.242	1.53	1	0.192	0.74	0.46	1.19
Others *	0.056	0.152	0.13	1		1.06	0.78	1.43
No Active Treatment	0.264	0.164	2.58	1		1.39	0.94	1.80
Albumin (g/dL)	−0.391	0.100	15.18	1	<0.001	0.68	0.56	0.82
Disease Stage	0.423	0.161	6.94	1	0.008	1.53	1.11	2.09
Metastatic								
Non-Metastatic								
ECOG Performance Status	1.230	0.130	91.32	1	<0.001	3.45	2.68	4.45
<2								
≥2								
Unplanned Hospital Readmission	0.260	0.118	4.88	1	0.027	1.30	1.03	1.63
Yes								
No								
Hemoglobin (Mean) (g/dL)	−0.025	0.27	0.86	1	0.35	0.98	0.93	1.03

* Tyrosine kinase inhibitor, immunotherapy, and antibody–drug conjugate. Eastern Cooperative Oncology Group: ECOG B: coefficients; S.E.: standard error; df: degrees of freedom; HR: hazard ratio; CI, 95%: confidence interval.

## Data Availability

The data that support the findings of this study are available from the corresponding author upon reasonable request.

## Supplementary Materials

The following supporting information can be downloaded at: https://www.mdpi.com/article/10.3390/jcm14082679/s1, Table S1: Cox regression analysis Assumption table; Table S2: Logistic regression analysis assumption table.
