# Supplementary material for: Clinical Characteristics and Management of Patients Admitted to the Supportive Care Clinic and Predisposing Factors of Unplanned Hospital Readmission: Single-Center Experience
Source: J Clin Med. 2025 Apr 14;14(8):2679. doi: 10.3390/jcm14082679 (PMC12028016; doi:10.3390/jcm14082679)
Supplement: Supplementary file 1 [file jcm-14-02679-s001.zip › jcm-3527151-supplementary.pdf]

Table S1. Cox regression analysis assumption table.

Assumption	Method Used	Comment
Non-Informative Censoring	Baseline comparison	Descriptive analysis showed similar baseline characteristics
Model Specification	Literature review	Covariates were selected based on the prior literature
Independence of Survival	No repeated measures	No clustering present
Proportional Hazards	Graphical checks	Survival plots showed parallel curves, supporting the proportional hazards assumption
Linearity	SPSS→ scatterplot/dot plot check	Scatterplot shows a random spread

Table S2. Logistic regression analysis assumption table.

Assumption	Method Used	Comment
Model Fit	Hosmer–Lemeshow Test	P = 0.462
Linearity of Continuous Variables	Scatterplot Check	Continuous variables showed no strong non-linearity
Multicollinearity	Variance Inflation Factor (VIF) and Tolerance	All VIF and tolerance values are acceptable<br>(e.g., disease stage VIF: 1.05; tolerance: 0.95)
Influential Points	Cook’s D (With SPSS)	No observations exceeded the standard thresholds (max: 0.026)
